# The relationship between injury-related anxiety and participation motivation: Insights from athletes across competitive levels

**DOI:** 10.12669/pjms.41.6.11798

**Published:** 2025-06

**Authors:** Canan Sayın Temur

**Affiliations:** 1 Canan Sayın Temur Department of Sports Management, Faculty of Sport Sciences, Ankara Yıldırım Beyazıt University, 06010, Ankara, Turkiye

**Keywords:** Athlete psychology, Gender differences, Injury-related anxiety, National team participation, Participation motivation, Sports motivation

## Abstract

**Objective:**

This study aimed to explore the relationship between injury-related anxiety and participation motivation in athletes, with a focus on demographic factors such as gender, age, national team status, and injury history.

**Methods:**

A cross-sectional design was employed, involving 221 athletes who completed validated scales measuring injury anxiety and participation motivation. Data were analyzed using t-tests, Mann-Whitney U tests, and correlation analyses.

**Results:**

Significant gender differences were found in anxiety subdimensions, with female athletes reporting higher levels of anxiety about loss of athleticism, pain, and reinjury. Age showed a negative correlation with anxiety about pain and reinjury, while positively correlating with motivational dimensions related to success, team membership, and physical fitness. National team athletes exhibited heightened anxiety related to social pressures and performance expectations. Athletes with an injury history reported elevated anxiety about pain and reinjury. Motivation subdimensions showed mixed relationships with anxiety subdimensions, with notable correlations between enjoyment, friendship, and anxiety dimensions.

**Conclusion:**

The findings emphasize the need for tailored psychological interventions and motivational strategies to address anxiety and enhance athletic performance across different demographic and experiential groups. These insights contribute to the growing literature on sports psychology and athlete well-being.

## INTRODUCTION

Sport is a vital means of mitigating the adverse effects of stress on metabolic processes, offering undeniable health benefits.^1^,^2^ However, as the number of athletes focusing intensely on specific sports increases, so does the level of competition.^3^ This growing emphasis on single-sport specialization, coupled with recent shifts in sports policies, has positioned sports as significant economic and political tools, profoundly affecting athletes’ lives.^4^,^5^ As a result, athletes are subjected to increasingly intense and frequent training sessions, which, despite their potential benefits, often lead to performance declines or sports injuries.^3^

Sports injuries are defined as damage resulting from excessive force applied to the body or a specific region beyond its tolerance limit.^6^ The nature of these injuries varies depending on the type of sport but can generally be categorized into bone, organ, or soft tissue injuries.^7^,^8^ The etiology of sports injuries is multifactorial, encompassing age, gender, physical fitness, psychomotor development, rehabilitation adequacy, warm-up routines, sport type, environmental and climatic conditions, training design, and activity duration.^9^ High-risk sports for injuries include football, wrestling, handball, boxing, skiing, and athletics.^10^

The consequences of sports injuries extend beyond physical damage, eliciting considerable psychological distress and anxiety among athletes. Injury-related anxiety is characterized by a heightened fear of getting injured or re-injured and concerns about its impact on performance and career.^11^ In sports contexts, anxiety can be defined as a decrease in self-assurance or an increased fear of failure due to perceived obstacles or unmet objectives, manifesting in both cognitive and somatic.^12^ Such injury-related anxiety can negatively influence athletes’ mental readiness, performance, and overall willingness to continue participating in sports.^11^ Injury-related anxiety can influence athletes’ mental readiness, performance, and overall participation in sports. Motivation-the driving force behind human behavior-is another critical factor intricately linked to injury-related anxiety.^12^

Motivation in sports is categorized as intrinsic, extrinsic, or amotivation. Intrinsic motivation is self-driven and arises from personal enjoyment or satisfaction, whereas extrinsic motivation is influenced by external rewards or stimuli. Amotivation reflects a lack of perceived connection between actions and outcomes, often resulting in disengagement.^13^ Understanding the dynamics of motivation is essential, as it not only drives athletic performance but also influences recovery and return to sports following injuries.

Despite recognition that injury-related anxiety can affect athletes’ motivation and performance, limited research has systematically explored this relationship. Existing studies tend to focus on either the psychological consequences of sports injuries or on general participation motivation, leaving a gap in understanding how these factors interact. Notably, few works have examined injury-related anxiety *in conjunction with* participation motivation across different athlete groups. To our knowledge, no prior study has investigated how injury-related anxiety correlates with motivation to participate in sports while considering demographic differences (such as gender and competitive level). The present study addresses this gap by examining the relationship between injury-related anxiety and participation motivation among athletes and by determining how this relationship may vary based on gender, age, elite athlete status, and injury history. By exploring these dynamics, this research offers a novel contribution to the sports psychology literature and provides practical insights for coaches, sports medicine professionals, and policymakers aiming to support athletes’ mental and physical well-being.

## METHODS

This study employed a cross-sectional design to examine the relationship between injury anxiety and motivation to participate in sports among athletes. A cross-sectional design was chosen to provide a snapshot of the variables at a specific point in time, enabling efficient data collection from a relatively large sample. Participants were informed about the study’s purpose and provided consent via an online Google Form, which also included the scales.

### Ethical approval:

This study was obtained from the Ankara Yıldırım Beyazıt University Ethics Committee (Approval number 01-569, dated January 16, 2024) before data collection. The study procedures were conducted in accordance with the principles of the Declaration of Helsinki. All research activities took place at Ankara Yıldırım Beyazıt University.

### Participants

The study population consisted of 221 athletes (53.8% male, 46.2% female) actively engaged in sports in Turkey. A convenience sampling approach was utilized due to its practicality in reaching participants within time and budget constraints. While this method facilitated data collection, it may introduce limitations in generalizability. Within the sample, 39.8% of participants were elite athletes (national team members) and 60.2% were non-national (sub-elite or recreational) athletes. A large majority (87.3%) had a history of at least one sports-related injury. Participants ranged in age from 12 to 72 years (mean age 22.87 ± 7.60 years). All participants were informed about the study’s purpose and provided informed consent via an online form. The data were collected through a secure online questionnaire (Google Forms) that included demographic questions and the study scales. The study was conducted in Ankara, Turkey, between April 15, 2024, and June 8, 2024, during which participants were instructed to complete the form individually in a quiet environment free from distractions to ensure consistency.

### Sports Injury Anxiety Scale (SIAS):

The SIAS, developed by Rex and Metzler (2016)^14^ and adapted into Turkish by Caz, Kayhan, and Bardakçı (2019)^1^, was used to measure injury-related anxiety. The scale comprises six subdimensions and 19 items, scored on a five-point Likert scale ranging from 1 (strongly disagree) to 5 (strongly agree). Higher scores indicate greater anxiety levels in each subdimension. The psychometric properties of the Turkish version have been validated in prior studies.

### Participation Motivation Questionnaire (PMQ):

The PMQ, developed by Gill, Gross, and Huddleston (1983)^15^ and, measures motivational factors influencing sports participation. The scale includes eight subdimensions and 30 items, scored on a three-point Likert scale. Lower scores indicate higher motivation levels. The validity and reliability of the scale were confirmed by Oyar et al. (2001).^16^

**Table- I:** Participant Demographics.

Variable	Category	n (%)
Gender	Male	119 (53.8)
	Female	102 (46.2)
National Team	Yes	88 (39.8)
	No	133 (60.2)
Injury History	Yes	193 (87.3)
	No	28 (12.7)
Age	Mean ± SD (Range)	22.87 ± 7.60 (12–72)

### Statistical Analysis:

Data were analyzed using SPSS 25. Normality was assessed using skewness and kurtosis values. Parametric tests were applied for normally distributed data, while non-parametric tests were used for non-normal distributions. T-tests and Mann-Whitney U tests were conducted for categorical variables, while Pearson correlation analysis was used for continuous variables. Statistical significance was set at p < 0.05.

## RESULTS

### Skewness and Kurtosis Analysis:

The subdimensions of the SIAS exhibited normal distributions with skewness and kurtosis values within the acceptable range (±2). However, most PMQ subdimensions showed non-normal distributions, suggesting the need for non-parametric analysis (Table-II).

**Table-II:** Skewness and Kurtosis Values for Scale Subdimensions.

Scale	Subdimension	Skewness	Kurtosis
SIAS	Anxiety About Losing Talent	0.406	-0.774
	Anxiety About Being Perceived as Weak	1.029	-0.281
	Anxiety About Suffering	-0.930	0.112
	Anxiety About Causing Disappointment	0.177	-1.157
	Anxiety About Losing Social Support	0.750	-0.943
	Anxiety About Re-Injury	-0.622	-0.530
PMQ	Achievement/Status	2.303	5.453
	Team Spirit/Membership	2.985	9.066
	Physical Fitness	1.831	3.263
	Enjoyment	1.876	2.709
	Friendship	1.820	2.819
	Competition	2.918	8.148
	Movement	3.214	9.502
	Skill Development	4.786	23.621

SIAS: Sports Injury Anxiety Scale. PMQ: Participation Motivation Questionnaire. Skewness: Indicates the asymmetry of the distribution of scores for each subdimension. Kurtosis: Indicates the “tailedness“ of the distribution. Normal distributions fall between ±2 for both skewness and kurtosis. Subdimensions with skewness or kurtosis beyond ±2 are considered non-normally distributed.

### Correlation Analysis Between Age and Subdimensions of SIAS and PMQ:

Significant correlations were identified between age and various subdimensions. For SIAS, age negatively correlated with Anxiety About Experiencing Pain (r = -0.205, p < 0.01) and Anxiety About Re-Injury (r = -0.173, p < 0.05). For PMQ, positive correlations were observed with Achievement/Status (r = 0.200, p < 0.01) and Team Membership (r = 0.174, p < 0.05) (Table-III).

**Table-III:** Correlation Analysis Between Age and Subdimensions of SIAS and PMQ.

Subdimension	Correlation Coefficient (r)	p-value
***SIAS***		
Anxiety About Losing Talent (ALA)	-0.120	0.076
Anxiety About Being Perceived as Weak (ABPW)	-0.050	0.461
Anxiety About Experiencing Pain (AEP)	-0.205**	0.002
Anxiety About Causing Disappointment (ALIO)	-0.021	0.751
Anxiety About Losing Social Support (ALSS)	0.072	0.289
Anxiety About Re-Injury (AR)	-0.173*	0.010
***PMQ***		
Achievement/Status	0.200**	0.003
Team Membership/Spirit	0.174**	0.010
Physical Fitness	0.164*	0.015
Enjoyment	0.097	0.150
Friendship	0.041	0.547
Competition	0.108	0.110
Movement	0.095	0.160
Skill Development	0.025	0.708

SIAS: Sports Injury Anxiety Scale. Correlation Coefficient (r): Indicates the strength and direction of the relationship between age and each SIAS subdimension. Negative values show an inverse relationship, while positive values show a direct relationship.

* p < 0.05;

** p < 0.01: Statistically significant correlations.

### T-Test Results for SIAS Subdimensions Based on Gender, National Team Status, and Injury History:

Gender differences were significant in Anxiety About Losing Talent (p = 0.022), Anxiety About Experiencing Pain (p = 0.013), and Anxiety About Re-Injury (p = 0.002), with females scoring higher. National team athletes reported higher anxiety levels in Anxiety About Disappointment (p = 0.000) and Anxiety About Social Support (p = 0.011). Similarly, athletes with injury history scored significantly higher in Anxiety About Experiencing Pain (p = 0.000) and Anxiety About Re-Injury (p = 0.002) (Table-IV).

**Table-IV:** T-Test Results for SIAS Subdimensions Based on Gender, National Team Status, and Injury History.

Subdimension	Comparison	Male	Female	t-value	p-value	Yes (NT)	No (NT)	t-value	p-value	Yes (Injury)	No (Injury)	t-value	p-value
ALA	Gender	7.75 ± 3.65	8.92 ± 3.87	-2.316	0.022*	8.82 ± 4.20	7.94 ± 3.47	1.628	0.105	8.42 ± 3.88	7.39 ± 3.06	1.341	0.181
ABPW	Gender	6.07 ± 3.72	6.96 ± 4.53	-1.585	0.115	6.83 ± 4.66	6.25 ± 3.74	0.980	0.329	6.54 ± 4.21	6.07 ± 3.55	0.559	0.577
AEP	Gender	10.79 ± 3.51	11.93 ± 3.23	-2.501	0.013*	11.72 ± 3.40	11.05 ± 3.43	1.143	0.159	11.65 ± 3.35	9.00 ± 3.07	3.958	0.000**
ALIO	Gender	8.24 ± 3.81	9.25 ± 3.88	-1.947	0.053	9.90 ± 4.15	7.91 ± 3.46	3.718	0.000**	8.89 ± 3.90	7.43 ± 3.44	1.874	0.062
ALSS	Gender	6.66 ± 4.16	7.55 ± 4.66	-1.480	0.140	8.05 ± 4.96	6.43 ± 3.89	2.580	0.011*	7.08 ± 4.54	7.00 ± 3.39	0.115	0.909
AR	Gender	13.39 ± 4.96	15.43 ± 4.50	-3.188	0.002**	15.07 ± 5.33	13.84 ± 4.46	1.784	0.076	14.66 ± 4.91	12.07 ± 3.77	3.252	0.002**

ALA: Anxiety About Losing Talent, ABPW: Anxiety About Being Perceived as Weak, AEP: Anxiety About Experiencing Pain, ALIO: Anxiety About Causing Disappointment, ALSS: Anxiety About Losing Social Support, AR: Anxiety About Re-Injury, NT: National Team

* p < 0.05;

** p < 0.01: Statistically significant differences.

### Mann-Whitney U Test Results for PMQ Subdimensions:

Significant differences in PMQ subdimensions were found for Physical Fitness (p = 0.019) and Enjoyment (p = 0.021) between national team and non-national team athletes, with national team athletes scoring higher (Table-V).

**Table V:** Mann-Whitney U Test Results for PMQ Subdimensions Based on Gender, National Team Status, and Injury History

Subdimension	Comparison	Male (Mean Rank)	Female (Mean Rank)	U-value	p-value	Yes (NT)	No (NT)	U-value	p-value	Yes (Injury)	No (Injury)	U-value	p-value
Achievement/Status	Gender	115.47	105.78	5536.5	0.177	106.80	113.78	5482.0	0.340	108.89	125.52	2295.5	0.123
Team Membership	Gender	109.32	112.96	5869.0	0.537	107.18	113.53	5516.0	0.291	110.71	113.02	2645.5	0.794
Physical Fitness	Gender	114.27	107.19	5680.0	0.329	100.55	117.91	4932.0	0.019*	108.75	126.52	2267.5	0.102
Enjoyment	Gender	110.92	111.10	6059.0	0.980	121.17	104.27	4957.0	0.021*	110.83	112.16	2669.5	0.902
Friendship	Gender	105.73	117.15	5442.0	0.130	119.07	105.66	5142.0	0.081	108.78	126.32	2273.0	0.120
Competition	Gender	112.33	109.45	5910.5	0.579	105.23	114.82	5344.0	0.070	109.85	118.93	2480.0	0.245
Movement	Gender	112.45	109.30	5896.0	0.491	108.32	112.77	5616.0	0.339	110.35	115.46	2577.0	0.455
Skill Development	Gender	112.33	109.45	5911.0	0.458	111.75	110.50	5786.0	0.752	111.01	110.93	2700.0	0.989

PMQ: Participation Motivation Questionnaire. NT: National Team.

* p < 0.05: Statistically significant differences.

### Correlation Analysis Between SIAS and PMQ Subdimensions:

Significant correlations emerged between anxiety and motivation subdimensions. Negative correlations were found between Anxiety About Losing Talent and Achievement/Status (r = -0.180, p < 0.05), while positive correlations were identified between Anxiety About Being Perceived as Weak and Enjoyment (r = 0.268, p < 0.01). These findings highlight the complex interplay between anxiety and motivational factors (Table-VI). The results highlight significant differences and correlations in SIAS and PMQ subdimensions based on age, gender, national team participation, and injury history. Gender differences revealed higher anxiety levels in females, while national team participation increased social-related anxieties. Injury history was significantly associated with higher “Anxiety About Re-injury” and “Anxiety About Experiencing Pain.” These findings emphasize the need for targeted psychological interventions and support for specific athlete groups.

**Table-VI:** Correlation Analysis Between SIAS and PMQ Subdimensions.

SIAS Subdimension	PMQ Subdimension	Correlation Coefficient (r)	p-value	Significance
Anxiety About Losing Talent (ALA)	Achievement/Status	-0.180	0.007*	Negative
	Physical Fitness	-0.172	0.011*	Negative
	Enjoyment	0.201	0.003*	Positive
	Friendship	0.135	0.045*	Positive
Anxiety About Being Perceived as Weak (ABPW)	Achievement/Status	-0.162	0.016*	Negative
	Enjoyment	0.268	0.000**	Positive
	Friendship	0.205	0.002**	Positive
Anxiety About Experiencing Pain (AEP)	Achievement/Status	-0.257	0.000**	Negative
	Team Membership/Spirit	-0.186	0.006*	Negative
	Physical Fitness	-0.175	0.009*	Negative
Anxiety About Causing Disappointment (ALIO)	Enjoyment	0.186	0.006*	Positive
Anxiety About Losing Social Support (ALSS)	Enjoyment	0.199	0.003*	Positive
	Friendship	0.198	0.003*	Positive
Anxiety About Re-Injury (AR)	Achievement/Status	-0.315	0.000**	Negative
	Physical Fitness	-0.200	0.003*	Negative
	Competition	-0.193	0.004*	Negative
	Skill Development	-0.193	0.004*	Negative

SIAS: Sports Injury Anxiety Scale. PMQ: Participation Motivation Questionnaire.

* p < 0.05;

** p < 0.01: Statistically significant correlations. Significance: Indicates the direction of the correlation (Positive or Negative)

## DISCUSSION

This study examined the psychological dimensions of injury-related anxiety and participation motivation among athletes, uncovering significant differences influenced by gender, age, national team participation, and injury history. Gender was found to significantly impact specific subdimensions of injury anxiety, including Anxiety About Loss of Athleticism (ALA), Anxiety About Experiencing Pain (AEP), and Anxiety About Reinjury (AR) (p < 0.05). These findings align with Güler^17^, who observed similar gender differences in AEP among handball players, and Tanyeri 10, who reported significant effects in ALA. However, the results also contrast with studies such as those by Karayol & Eroğlu 6 and Contarlı & Ozmen^18^, which found no significant gender-based differences in SIAS subdimensions. Such discrepancies may be attributable to differences in sample characteristics or sporting contexts. Female athletes in our sample, who spanned various sports and competitive levels, might face distinct pressures or cultural expectations that heighten certain anxieties (for instance, concerns about bodily harm or performance failure). These mixed findings across studies warrant further exploration into how gender roles and support systems influence injury-related fears. On a broader scale, our observation that female athletes exhibit higher injury-related anxiety aligns with trends reported in the wider literature. A recent meta-analysis 19 on competitive anxiety in athletes concluded that female gender and younger age are associated with higher anxiety levels. This meta-analytic evidence suggests that gender differences in sport-related anxiety are a robust phenomenon observed across diverse competitive settings Thus, our study’s gender-related findings not only mirror local research but also resonate with established patterns internationally, reinforcing the importance of addressing female athletes’ psychological needs.

The relationship between age and injury anxiety yielded intriguing insights, with older athletes exhibiting lower levels of AR and AEP. This finding suggests that with increasing age (and presumably experience), athletes may develop better coping strategies, greater confidence in managing injuries, or a more realistic outlook on injury risks, which together confer a protective effect against anxiety. Nevertheless, prior studies, such as those by Bağrıacık & Açak (2005)^8^ and Namlı & Buzdağlı (2020)^20^, reported positive correlations between age and AR, indicating that the relationship between these variables may depend on contextual factors such as sport type, competition level, and athlete experience.

National team athletes demonstrated significantly higher levels of Anxiety About Letting Down Important Others (ALIO) and Anxiety About Loss of Social Support (ALSS) compared to their non-national counterparts (p < 0.05). These findings highlight the elevated psychological pressures faced by athletes representing their countries, including fears of disappointing significant others and losing social connections. Such pressures likely stem from the heightened expectations and scrutiny associated with elite-level competition. Supporting this observation, Öner (2023)^21^ reported similar trends among national team athletes, although Ökmen and Sarıkaya (2023)^22^ identified significant differences primarily in ALA and AEP among amateur and professional taekwondo athletes. These variations may underscore the moderating effects of sport-specific and competitive contexts.

The study also revealed that athletes with a history of injuries reported higher levels of AEP and AR (p < 0.05), reflecting the enduring psychological impact of past injury experiences. This finding aligns with research by Öner (2023)^21^, who established a strong association between injury history and AEP highlighted elevated anxiety levels among previously injured athletes. The findings underscore the need for targeted psychological support for this population, particularly interventions addressing fear of reinjury and pain anticipation.

Participation motivation, as assessed through the PMQ, exhibited varying relationships with demographic factors. Gender differences in PMQ subdimensions were not statistically significant (p > 0.05), consistent with findings by Akdağ & Türkmen (2023)^23^ and Karaç (2017).^24^ However, contrasting results in studies by Aycan & Yıldız (2016)^25^ and Ryan et al. (1990)^26^ suggest that contextual factors such as cultural norms and training environments may influence motivational patterns. Age, on the other hand, was positively correlated with PMQ subdimensions such as Success, Team Membership/Spirit, and Physical Fitness (p < 0.05). This indicates that older athletes derive greater motivation from their sense of achievement and collective goals, possibly as a compensatory mechanism for physical and social changes associated with aging. These findings suggest that age-related shifts in motivation warrant further investigation to better understand their underlying mechanisms.

The interplay between injury anxiety and participation motivation revealed complex dynamics. Most injury anxiety subdimensions exhibited negative correlations with performance-oriented motivations such as Success and Physical Fitness, highlighting the inhibitory effects of anxiety on achievement-related goals. However, positive correlations were observed between certain anxiety subdimensions and Enjoyment and Friendship, suggesting that specific forms of anxiety may foster camaraderie and collective coping among athletes. These findings add nuance to the understanding of how psychological stressors interact with motivational factors in athletic contexts.

### Limitations:

This study provides meaningful insights into the relationship between injury-related anxiety and motivation; however, some limitations must be acknowledged. The cross-sectional design prevents causal interpretation—whether anxiety leads to motivational changes or vice versa remains unclear. Reliance on self-report tools may also introduce bias, and the convenience sampling limits generalizability beyond the studied group, which was predominantly young and previously injured. Additionally, sport-specific variations were not analyzed, which could influence psychological responses.

Future research should consider longitudinal designs to explore temporal dynamics and potential causality. Incorporating objective or qualitative methods may deepen understanding, while intervention-based studies could test strategies to reduce injury anxiety or enhance motivation. Broader and more diverse samples would help determine the universality of these findings across different sports and cultural settings.

## CONCLUSION

This study advances our understanding of the interplay between injury-related anxiety and participation motivation in athletes, emphasizing how personal and situational factors shape this relationship. Key findings indicate that female and younger athletes tend to experience higher injury anxiety, elite athletes face distinct social-pressure anxieties, and previously injured athletes remain particularly anxious about pain and reinjury. Meanwhile, athletes’ motivations to engage in sport can shift in response to these anxieties – often de-emphasizing competitive achievement while leaning more on enjoyment and social aspects when anxiety is high.

Collectively, the findings underscore the importance of tailored interventions that consider individual differences in gender, age, competitive level, and injury history. Sports organizations and professionals should recognize that psychological factors like anxiety can significantly impact an athlete’s motivation and performance. By addressing these psychological dimensions – for instance, through mental skills training, anxiety management programs, and fostering a supportive team environment – coaches and practitioners can help athletes maintain a healthy balance of motivation and anxiety. Ultimately, such efforts contribute to the holistic development and sustained well-being of athletes, fostering resilience in the face of injuries and enhancing their long-term engagement and success in sport.
